# Perceived morbidity and community burden after a Chikungunya outbreak: the TELECHIK survey, a population-based cohort study

**DOI:** 10.1186/1741-7015-9-5

**Published:** 2011-01-14

**Authors:** Patrick Gérardin, Adrian Fianu, Denis Malvy, Corinne Mussard, Karim Boussaïd, Olivier Rollot, Alain Michault, Bernard-Alex Gaüzere, Gérard Bréart, François Favier

**Affiliations:** 1 Centre for Clinical Investigation-Clinical Epidemiology (CIC-EC) of La Réunion (INSERM/CHR/URMLR), Saint Pierre, La Réunion, France; 2 Neonatal and Pediatric Intensive Care Unit, Centre Hospitalier Régional (CHR), Saint Pierre, La Réunion, France; 3 UMR S953, 'Epidemiological Research on Perinatal Health and Women and Children Health' (INSERM/Assistance Publique des Hôpitaux de Paris), Paris, France; 4 Department of Internal Medicine and Tropical Diseases, Hôpital Saint André, Bordeaux, France; 5 Microbiology, CHR, Saint Pierre, La Réunion, France; 6 Polyvalent Intensive Care Unit, CHR, Saint Denis, La Réunion, France; 7 Public Health Institute, INSERM, Paris, France

## Abstract

**Background:**

Persistent disabilities are key manifestations of Chikungunya virus (CHIKV) infection, especially incapacitating polyarthralgia and fatigue. So far, little is known about their impact on health status. The present study aimed at describing the burden of CHIKV prolonged or late-onset symptoms on the self-perceived health of La Réunion islanders.

**Methods:**

At 18 months after an outbreak of Chikungunya virus, we implemented the TELECHIK survey; a retrospective cohort study conducted on a random sample of the representative SEROCHIK population-based survey. A total of 1,094 subjects sampled for CHIKV-specific IgG antibodies in the setting of La Réunion island in the Indian Ocean, between August 2006 and October 2006, were interviewed about current symptoms divided into musculoskeletal/rheumatic, fatigue, cerebral, sensorineural, digestive and dermatological categories.

**Results:**

At the time of interview, 43% of seropositive (CHIK+) subjects reported musculoskeletal pain (vs 17% of seronegative (CHIK-) subjects, *P *< 0.001), 54% fatigue (vs 46%, *P *= 0.04), 75% cerebral disorders (vs 57%, *P *< 0.001), 49% sensorineural impairments (vs 37%, *P *= 0.001), 18% digestive complaints (vs 15%, *P *= 0.21), and 36% skin involvement (vs 34%, *P *= 0.20) on average 2 years after infection (range: 15-34 months). After controlling for confounders such as age, gender, body mass index or major comorbidities in different Poisson regression models, 33% of joint pains were attributable to CHIKV, 10% of cerebral disorders and 7.5% of sensorineural impairments, while Chikungunya did not enhance fatigue states, digestive and skin disorders.

**Conclusions:**

On average, 2 years after infection 43% to 75% of infected people reported prolonged or late-onset symptoms highly attributable to CHIKV. These manifestations carry a significant burden in the community in the fields of rheumatology, neurology and sensorineural health.

## Background

Chikungunya virus (CHIKV) is an enveloped RNA positive-strand alphavirus belonging to the *Togaviridae *family, transmitted by *Aedes *mosquitoes [1]. CHIKV is now known to target human epithelial and endothelial cells, fibroblasts and macrophages [2], human muscle satellite cells [3], and to cause a wide range of acute manifestations including fever, arthralgia, myalgia, rash and fatigue [4,5]. Furthermore, CHIKV infection often leads to prolonged joint pain, whose stability or relapses characterize the hallmark symptom of 'Chikungunya rheumatism' [5-7]. The basis of musculoskeletal pain and chronic arthropathy observed after acute CHIKV infection may come from the early escape of CHIKV from blood monocytes [8], allowing its relocation to synovial macrophages, as seen with the Ross River virus (RRV) [9], rather than from autoimmunity. The persistence of CHIKV in such sanctuaries triggers a sustained low-noise T helper 1 cell (Th1) response leading to chronic inflammation and fibrosis, as shown by recent findings in a non-human primate model [10]. Such a pathway has been previously outlined by a single case report and subsequently confirmed by a large case series, both from La Réunion Island infected individuals [11,12].

Beyond these common features, CHIKV infection also exhibits more atypical forms [13,14]. These include cerebral disorders resulting from a now well established neurotropism [15,16], ranging from mild disrupted behavior or altered mental status to severe encephalopathy/encephalitis [16-19] and to various sensorineural impairments [20,21]. Concurrently, CHIKV infection is known to account for a broad spectrum of mucocutaneous lesions [22,23]. Interestingly, investigation of the pathogenesis, natural course and clinical impact has so far been neglected.

These prolonged or late-onset symptoms occur either at the acute stage or during convalescence and their clinical picture is neither specific nor consistent between individuals. Whether these manifestations are directly attributable to CHIKV infection or represent the exacerbation or complications of underlying conditions, such as osteoarthritis [7], or account for the mode of entry of a subsequent chronic disease, such as rheumatoid arthritis (RA) or psoriatic rheumatism [24,25], is still unknown. Moreover, their clinical burden for the community has not been researched beyond a small and non-representative matched cohort study [26].

To address these important topics, we designed the TELECHIK survey, a telephonic interview through the framework of the SEROCHIK survey. The goal of the SEROCHIK survey was to clarify the seroprevalence and the risk factors of CHIKV infection within the community of La Réunion in the context of the large-scale epidemic that swept the island in 2005-2006 [27,28]. The current study assesses the self-perceived morbidity by La Réunion islanders but also reveals the public health impact of Chikungunya disease in showing the true areas of the community burden, on average 18 months after the outbreak incident.

## Methods

### Study design and population

Participants were enrolled in a retrospective cohort study, the TELECHIK survey, designed from the framework of the cross-sectional, population-based SEROCHIK survey [27,28]. The latter, conducted in La Réunion between 17 August and 20 October 2006 soon after an outbreak of Chikungunya virus (June 2006), involved a representative sample of 2,442 index individuals for whom their medical history in relation to Chikungunya was sought and a specific serological enzyme-linked immunosorbent assay (ELISA) test performed [27].

Exposure to CHIKV was defined as positive for the subjects with positive CHIKV-specific IgG antibodies. Seropositive (CHIK+) and seronegative (CHIK-) subjects were allocated by classifying the previous exposure item within six strata defined at the time of the SEROCHIK survey as follows: true positive (TP, symptomatic CHIK+), false negative (FN, asymptomatic CHIK+), not knowing positive (NKP, CHIK+ without memories of symptoms and serostatus), true negative (TN, asymptomatic CHIK- negative), false positive (FP, symptomatic CHIK-), not knowing negative (NKN, CHIK- without reminiscence of symptoms and serostatus) in order to account for the declaration bias beyond the representativeness of our cohort. Taking into account a feasibility constraint, two subsets of the same size of TP and TN subjects were randomly selected after stratification for age, gender and area of residence, with the aim to control the repartition bias. This allocation was conducted by applying reasoned sampling fractions (TP: 0.7 and TN: 0.46), while FP, NKP, FN, NKN, and fewer at SEROCHIK inclusion, were all systematically selected.

### Setting

La Réunion is a French overseas 'département' of 805,500 inhabitants, located on a volcanic island of 2,511 km^2 ^belonging to the Mascarene archipelago in the southwestern Indian Ocean. In 2005-2006, La Réunion was, for the first time in its history, overwhelmed by a Chikungunya epidemic of unprecedented magnitude. More than 266,000 clinical cases were noted by the public health authorities and nearly 300,000 inhabitants (38.2%) were considered to have been infected [27]. The main reason for such widespread emergence of CHIKV was a single mutation in the envelope protein gene (E1-A226V) that increased the fit of the virus for its principal vector, *Aedes albopictus*, the Asian tiger mosquito [29,30]. Furthermore, *Ae albopictus*, was known to exhibit an unusual adaptive property to the human host (so-called anthropization) in La Réunion for a period [31], which was later highlighted by the recent entomological surveys [32].

### Data collection

Participants were interviewed by telephone between November 2007 and May 2008. A short questionnaire was administered by one blinded investigator (CM) to ensure the best possible reproducibility. It was composed of closed questions addressing the most currently reported symptoms. The latter accounted for musculoskeletal pain, headache, digestive disorders, sleeping disorders, memory troubles, attention difficulties, mood disturbance, depression, blurred vision, hearing difficulties, skin lesions, and alopecia.

Body mass index (BMI) was calculated as weight (kg) divided by the square of height (m^2^). Weight and height, as all the relevant comorbidities, were reported from the SEROCHIK survey. Parents or legal guardians were interviewed for persons aged under 15 years.

### Sample size determination and statistical analysis

The sample size was determined to enable the detection of a minimum 10% difference between infected (CHIK+) TP and uninfected (CHIK-) healthy TN subjects in musculoskeletal pain frequency, assuming a one-sided type I error of 5% and a power of 90%. The more conservative scenario, depicting a baseline incidence of musculoskeletal pain between 40% and 50% in the adult TN population, as suggested by those observed in the general UK population [33], and between 50% and 60% in the TP adult population (CHIK+ patients), required 560 TP, 560 TN, all the FP, NKP, FN, and NKN subjects, assuming a loss level of 33%, due to incomplete data plus non-responders.

Assessment of current symptoms according to explanatory variables was conducted by bivariate analysis. Proportions were compared using corrected design-based χ^2 ^tests and means using the adjusted Wald test as appropriate. Symptoms were categorized as musculoskeletal/rheumatic, fatigue, light cerebral (headache, and/or sleep disorders, memory troubles, attention difficulties, mood disturbance, depression), sensorineural (blurred vision, hearing difficulties), digestive (digestive disorders) and dermatological (skin lesions, alopecia). Crude prevalence ratios (cPR) and 95% confidence intervals (95% CIs) of CHIKV infection were generated using Poisson regression models for each symptom and category. The crude etiology fraction (EF) and 95% CI of CHIKV infection was calculated for each category linked to Chikungunya to assess the accountability of CHIKV in the genesis of an area of symptoms, as follows: EF (%) = (π_1 _- π_0_/π_1_) × 100, with π_1 _the prevalence of the symptom among CHIK+ subjects and π_0: _the prevalence of the symptom among CHIK- subjects. Adjusted prevalence ratios (aPR) and 95% CIs for Chikungunya were calculated in Poisson regression models controlling major confounders such as age, gender, BMI, and comorbidities. The population attributable risk (PAR) of CHIKV infection was calculated to assess the community burden of Chikungunya within the perceived morbidity on a population basis. To account for a possible subjectivity bias inherent of FP, NKP, FN, and NKN subjects, we did a sensitivity analysis in the sample restricted to TP and TN. To determine whether the knowledge of previous exposure may affect self-perceived morbidity later on follow-up, we also compared the declaration of each symptoms within FP and FN subsets, assuming that the subjectivity inherent to FP be expressed steadily over time. Finally, we tested the hypothesis that for CHIK+ TP subjects, a previous declaration of intense symptoms (defined as reports of fever plus arthralgia and/or myalgia at the onset of infection) was associated with a more frequent symptoms declaration on follow-up, assuming that subjectivity would not change the expected gradient of declarations from TN to intense TP (TN < mild TP < intense TP). For all theses analyses, we took special care to verify that the timing of questioning was similar across the groups, and the multiple stage complex sampling procedure was taken into account. Statistical significance was set at *P *= 0.05. EpiData software (v.3.1; EpiData, Copenhagen, Denmark) was used for data entry and Stata (v.10.0; StataCorp, College Station, TX, USA) for the analysis.

### Ethical considerations

During the SEROCHIK survey, which had previously received ethical approval from the ethical committee for studies with human subjects (CPP) of Bordeaux and the National Commission for Informatics and Liberty (CNIL) [27], the participants had been informed that they might be called back for ancillary research [26]. During the telephone interview, the objectives of the TELECHIK survey were presented and oral consent to participate was obtained.

## Results

**Figure 1:**
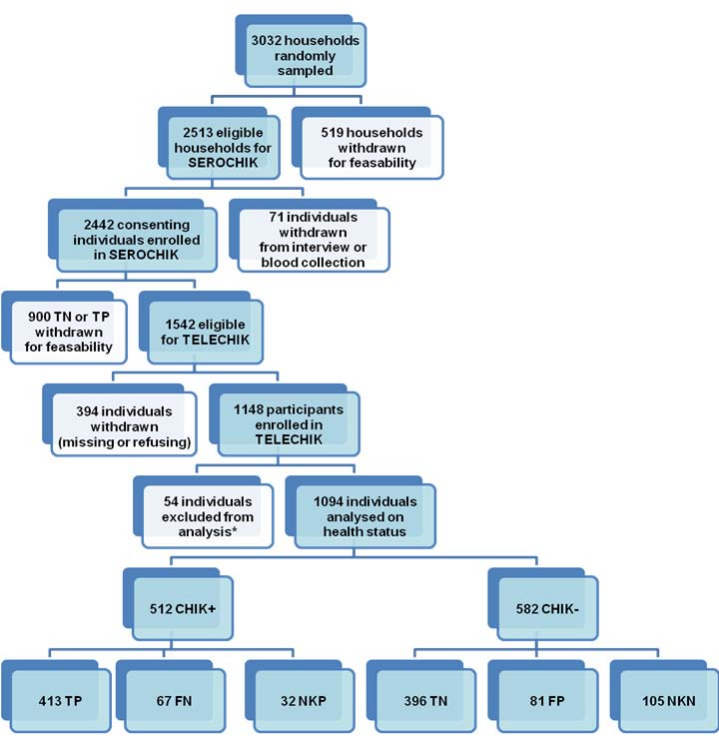
**Study participation profile of the TELECHIK cohort study**. Exposure was defined using Chikungunya virus (CHIKV)-specific IgG antibodies and self-report of common symptoms within six strata: FN = false negative, FP = false positive, NKN = not knowing negative, NKP = not knowing positive, TN: true negative, TP: true positive. *In all, 8 questionnaires were excluded for poor quality responses (impossibility of interpretation), and 46 were excluded for responders different from the index individual, parents or legal guardian in the TELECHIK survey.

Among the 1,542 individuals selected, 5 had died since the SEROCHIK survey (no death certificate mentioned Chikungunya as the leading cause for death; source: cepiDC, INSERM, France), 359 had missing contact details, 20 refused to answer the questionnaire and 54 were excluded from the analysis because of incomplete data or mismatched responders (different from the index person, parents, legal guardian); thus, 1,094 index individuals were retained for analysis (Figure 1).

The mean age of the participants was 36 years (range: 1 month to 93 years); 39% of subjects were aged less than 30 years, 48% were 30 to 59 years and 13% were 60 years or older. The male to female ratio was 0.74. The distribution of the area of residence was representative of the one observed in the community (Table 1). More females were participants compared to non-participants (59.0% vs 52.7%, *P *= 0.02), while age and residential area did not differ.

**Table 1:** Characteristics of the 1,094 subjects analyzed for current health status at follow-up TELECHIK survey, November 2007 to May 2008, and of La Réunion Island population

Characteristics, n (%)	TELECHIK	**INSEE**^**a**^
Gender:		

Women	645 (57.4)	414,855 (51.5)^b^

Men	449 (42.6)	390,645 (48.5)

Age, years:		

<20	214 (27.6)	281,680 (35.0)^b^

20 to 29	124 (11.8)	106,277 (13.2)

30 to 39	159 (16.3)	118,412 (14.7)

40 to 49	180 (17.6)	123,616 (15.3)

50 to 59	187 (14.2)	84,122 (10.4)

60 to 69	133 (7.7)	49,397 (6.1)

≥70 years	97 (4.8)	41,996 (5.2)

Residential area:^c^		

North	249 (22.8)	190,625 (24.4)^d^

East	167 (15.3)	114,278 (14.6)

South	403 (36.8)	277,602 (35.5)

West	275 (25.1)	199,457 (25.5)

^a ^Institut National de la Statistique et des Etudes Economiques; ^b^2008 census; ^c^La Réunion Island is divided into four administrative microregions: North, East, South, and West; ^d^2006 census.

Overall, following a mean time of 16 months from the SEROCHIK survey (range: 13-20 months, on average 15.93 months for CHIK+ vs 15.97 months for CHIK-, *P *= 0.60), 80.9% of the 1,094 participants interviewed for the TELECHIK survey reported 1 or more symptoms. The time elapsed between infection and telephone interview in CHIK+ subjects is given in Additional file 1.

Among infected individuals, the 413 TP subjects (median time between infection and interview: 23.7 months, Q_1_-Q_3_: 21.9-24.9 months, mode: 25 months, range: 15-34 months) did not differ from 67 FN and 32 NKP subjects on age, gender, BMI and major comorbidities, which allowed the consolidation of the 3 groups into 1 of 512 CHIK+ subjects. Similarly, among uninfected peers, even though 396 TN subjects were younger than 81 FP or 105 NKN subjects, and FP subjects more likely to declare several comorbidities, TN, FP, and NKN subjects were also regrouped into 1 group (CHIK-) to simplify the comparisons.

The crude PRs and 95% CIs for CHIKV infection for the current self-reported symptoms and categories of symptoms are shown in Table 2. CHIK+ subjects were more likely than CHIK- peers to complain of musculoskeletal pain (43% vs 17%, Crude PR: 2.5, 95% CI 1.9 to 3.1) with a high proportion attributable to CHIKV infection (EF: 60.0%, 95% CI 51.8% to 68.2%). CHIK+ subjects also more frequently reported fatigue, light cerebral disorders, and sensorineural impairment than their CHIK- peers. The EF of CHIKV infection was 13.5% (95% CI 3.2% to 25.8%) for fatigue, 24.2% (95% CI 17.7% to 30.8%) for light cerebral disorders and 23.8% (95% CI 13.3 to 34.3%) for sensorineural impairment.

**Table 2:** Crude weighted rates and prevalence ratios of self-reported symptoms/CHIKV status, TELECHIK survey, La Réunion Island population, November 2007 to May 2008

Symptoms (%)	Seronegative (CHIK-), %	Seropositive (CHIK+), %	Crude prevalence ratio	95% CI	*P *value
Musculoskeletal pain	17.1	42.8	2.5	1.9 to 3.1	<0.001

Fatigue	46.4	53.6	1.1	1.0 to 1.3	0.04

Light cerebral disorders	57.0	75.3	1.3	1.1 to 1.5	<0.001

Headache	20.0	25.9	1.3	1.0 to 1.7	0.05

Sleep disorders	24.1	31.2	1.3	1.0 to 1.7	0.02

Memory troubles	25.4	42.2	1.7	1.3 to 2.0	<0.001

Attention difficulties	19.9	37.1	1.9	1.4 to 2.3	<0.001

Mood disturbance	23.7	38.4	1.6	1.3 to 2.0	<0.001

Depression	8.0	14.7	1.8	1.2 to 2.7	0.002

Sensorineural disorders	37.2	48.8	1.4	1.1 to 1.5	0.001

Blurred vision	30.2	42.0	1.4	1.1 to 1.7	<0.001

Hearing difficulties	13.0	17.8	1.4	0.9 to 1.9	0.05

Digestive disorders	15.0	18.3	1.2	0.8 to 1.7	0.21

Dermatological disorders	34.2	36.1	1.2	0.8 to 1.7	0.205

Skin lesions	17.2	19.9	1.2	0.8 to 1.5	0.33

Alopecia	21.4	21.9	1.0	0.7 to 1.3	0.835

The weighted rates of the symptoms are expressed as percentages. Chikungunya status is defined by specific anti-CHIKV IgG antibodies.

Digestive and skin disorders (including alopecia) were not associated with Chikungunya infection. Among the light cerebral disorders, the most closely linked to CHIKV infection were attention difficulties (EF: 46.3%, 95% CI 35.7% to 57.0%), memory trouble (EF: 39.9%, 95% CI 29.5% to 50.2%), mood disturbance (EF: 38.3%, 95% CI 27.0% to 49.5%) and depression (EF: 45.6%, 95% CI 26.8% to 64.4%), whereas the association with headache (EF: 22.6%, 95% CI 5.7% to 39.5%) and sleep disorders (EF: 22.9%, 95% CI 8.0% to 37.8%) was slight. Among sensorineural impairments, Chikungunya infection affected vision (blurred vision, EF: 28.2%, 95% CI 16.7% to 39.7%) more than hearing (hearing difficulties, EF: 26.7%, 95% CI 6.2% to 47.3%). Consistently, the sensitivity analysis restricted to TP and TN (Additional file 2) depicted the same figures with stronger links, which argued for a causative role of CHIKV in the pathogenesis of symptoms (this time including hearing loss, *P *= 0.016), and indirectly for subjectivity in FP, NKP, FN, and NKN subsets. Against all odds, the FP subjects tended to declare almost every symptom more frequently than the FN subjects on follow-up, which argued for influence of subjectivity rather than knowledge of exposure in self-perceived morbidity at a distance. As expected, the TP subjects with the more intense symptoms at onset of infection complained slightly more frequently on follow-up than mild TP subjects, and far more than TN subjects, confirming the link between intense inaugural symptoms and late manifestations, as the minor role of subjectivity in CHIK+ subjects.

The adjusted PRs and 95% CIs inherent of CHIKV infection and major confounders for determining musculoskeletal/rheumatic pain, light cerebral disorders, and sensorineural impairment are shown in Tables 3, 4, 5, 6.

**Table 3:** Adjusted prevalence ratios for the determinants of self-reported rheumatic symptoms, TELECHIK survey, La Réunion Island population, November 2007 to May 2008

Determinants	Adjusted prevalence ratio	95% CI	*P *value
Chikungunya:			<0.001

No	1		

Yes	2.1	1.7 to 2.7	

Gender:			0.08

Male	1		

Female	1.2	0.9 to 1.5	

Age, years:			<0.001

<20	1		

20 to 29	1.4	0.7 to 2.5	

30 to 39	1.8	1.1 to 3.0	

40 to 49	2.7	1.7 to 4.2	

50 to 59	3.3	2.1 to 5.2	

60 to 69	3.9	2.5 to 6.1	

≥70	3.5	2.2 to 5.6	

Body mass index, kg/m^2^:			0.001

<25	1		

25-29.9	1.5	1.2 to 1.8	

≥30	1.3	0.9 to 1.7	

Comorbidity:^a^			0.11

None	1		

Osteoarthritis	1.4	1.0 to 2.0	

Other^a^	1.2	0.9 to 1.5	

^a ^Diabetes mellitus, hypertension, ischemic heart disease, asthma, chronic obstructive pulmonary disease, renal failure, cancer.

**Table 4:** Adjusted prevalence ratios for the determinants of fatigue, TELECHIK survey, La Réunion Island population, November 2007 to May 2008

Determinants	Adjusted prevalence ratio	95% CI	*P *value
Chikungunya:			0.11

No	1		

Yes	1.1	0.9 to 1.3	

Gender:			<0.001

Male	1		

Female	1.4	1.1 to 1.6	

Age, years:			0.003

<20	1		

20 to 29	1.7	1.2 to 2.2	

30 to 39	1.5	1.1 to 2.0	

40 to 49	1.5	1.1 to 2.0	

50 to 59	1.5	1.1 to 2.0	

60 to 69	1.4	1.0 to 1.8	

≥70	1.7	1.2 to 2.3	

Comorbidity:^a^			0.006

None	1		

1	1.2	1.0 to 1.5	

2	1.1	0.8 to 1.4	

≥3	1.5	1.2 to 1.8	

^a ^Osteoarthritis, diabetes mellitus, hypertension, ischemic heart disease, asthma, chronic obstructive pulmonary disease, renal failure, cancer.

**Table 5:** Adjusted prevalence ratios for the determinants of light cerebral disorders, TELECHIK survey, La Réunion Island population, November 2007 to May 2008

Determinants	Adjusted prevalence ratio	95% CI	*P *value
Chikungunya:			<0.001

No	1		

Yes	1.3	1.1 to 1.4	
Gender:			0.002

Male	1		

Female	1.2	1.0 to 1.3	

Age, years:			0.83

<20	1		

20 to 29	1.1	0.8 to 1.3	

30 to 39	0.9	0.8 to 1.2	

40 to 49	1.0	0.8 to 1.2	

50 to 59	1.1	0.9 to 1.3	

60 to 69	1.0	0.8 to 1.2	

≥70	0.9	0.7 to 1.2	

Comorbidity:^a^			0.04

None	1		

1	1.1	0.9 to 1.3	

2	1.2	1.0 to 1.4	

≥3	1.2	0.9 to 1.6	

^a ^Osteoarthritis, diabetes mellitus, hypertension, ischemic heart disease, asthma, chronic obstructive pulmonary disease, renal failure, cancer.

**Table 6:** Adjusted prevalence ratios for the determinants of sensorineural impairment, TELECHIK survey, La Réunion Island population, November 2007 to May 2008

Determinants	Adjusted prevalence ratio	95% CI	*P *value
Chikungunya:			0.02

No	1		

Yes	1.2	1.0 to 1.4	

Gender:			0.36

Male	1		

Female	1.1	0.9 to 1.3	

Age, years:			<0.001

<20	1		

20 to 29	1.7	1.0 to 2.5	

30 to 39	1.4	0.9 to 2.1	

40 to 49	2.6	1.9 to 3.6	

50 to 59	2.7	1.9 to 3.7	

60 to 69	2.7	1.9 to 3.8	

≥70	2.6	1.7 to 3.7	

Comorbidity:^a^			0.20

None	1		

1	1.1	0.9 to 1.3	

2	1.2	0.9 to 1.5	

≥3	1.3	0.9 to 1.6	

^a ^Osteoarthritis, diabetes mellitus, hypertension, ischemic heart disease, asthma, chronic obstructive pulmonary disease, renal failure, cancer.

When adjusting for major confounders, on average 24 months (range: 15-34 months) after the acute stage of the infection CHIK+ subjects were twice as likely to report musculoskeletal pain (aPR: 2.1, 95% CI 1.7 to 2.7) than their CHIK- counterparts (Table 3). Furthermore, they complained more easily of light cerebral disorders (aPR: 1.3, 95% CI 1.2 to 1.4) (Table 5). Similarly, they were also slightly associated with sensorineural impairment (aPR: 1.2, 95% CI 1.0 to 1.4) (Table 6). Accordingly, the same models applied to TP and TN subjects (Additional files 3, 4, 5) drew similar conclusions favoring the role of CHIKV, except those dedicated to fatigue. Fatigue was not enhanced in CHIK+ subjects compared with CHIK- peers (aPR: 1.1, 95% CI 0.9 to 1.3) when considering the whole sample (Table 4), whereas it was linked to infection (aPR: 1.2, 95% CI 1.0 to 1.4, *P *= 0.025) in TP and TN subjects (Additional file 6).

Importantly, the PARs for Chikungunya to explain musculoskeletal pain, light cerebral disorders and sensorineural impairment, as expressed by the community, were respectively 33.6%, 10.4% and 7.5%. In other words, if Chikungunya had been fully preventable, participants should have reported two-thirds of the musculoskeletal pains, 89.6% of the light cerebral disorders and 92.5% of the sensorineural impairments declared in the TELECHIK survey.

## Discussion

Here, we report a large follow-up study dealing with the community burden of Chikungunya long-term manifestations, experienced within the La Réunion population after the 2005-2006 Chikungunya virus outbreak [6,7,12]. One of the major findings of our population-based survey is that self-perceived morbidity, described through several subjective symptoms and categories of manifestations (musculoskeletal pain, fatigue, light cerebral disorders, sensorineural impairment, digestive and skin disorders), on average 18 months after the outbreak, is still largely attributed to Chikungunya. Thus, among previously exposed individuals (CHIK+ subjects), CHIKV would be involved in 60% of musculoskeletal pains (characterized indistinctively as joint, bone or muscle aches), approximately a quarter of cerebral disorders and of sensorineural impairments, and 13% of fatigue states. Moreover, at the general population level, after controlling major confounders, Chikungunya could explain a third of rheumatic symptoms, 10% of neurological and 7.5% of sensorineural complaints. These features highlight a previously misrecognized impact of CHIKV infection in the fields of rheumatology, neurology and sensorineural health.

To the best of our knowledge, beyond a smaller non-representative matched cohort study [26], no comparative study to date had attempted to establish the responsibility of Chikungunya for the persistence (or the delayed onset) of non-specific common symptoms, nor its public health impact. To address these questions we designed the TELECHIK study, a comparison set beyond the framework of the population-based SEROCHIK survey that allowed the selection of a random sample of the La Réunion island community [27,28]. Moreover to enable a random homogeneous allocation of subjectivity determinants of the symptoms reported, we also adjusted for major confounders of health status in order to assess the proper role of CHIKV infection in perceived morbidity. These restrictions, both in design and analysis, were necessary to make a causal relationship plausible between CHIKV and its related manifestations. Indeed, they are now recognized as an indisputable prerequisite to improve the standard of proof of observational studies [34].

The reality of 'Chikungunya rheumatism' was suggested formerly in a few case report series [35-37]. It has also been supported in recent years by several observational studies [4-7,38,39], whose design should not have definitely attributed such non-specific symptoms to CHIKV. For instance, in Transvaal, South Africa, 15% of patients complained of persistent arthralgia 20 months after acute infection [35], while 12% reported recurrent relapsing joint pain, stiffness or swelling 3 to 5 years after Chikungunya onset [36]. Nonetheless, these assessments were not conducted with any formal evaluation of the community burden of such symptoms. In contrast, cases imported to France from La Réunion exhibited a heavier burden with rheumatic manifestations of at least 48% 6 months to 2 years after the acute infection [38,39]. Of note, among La Réunion islanders, the persistence of rheumatic pain was perceived to be at a similarly alarming level, with 57% of CHIK+ subjects from a non-representative sample of the population experiencing permanent or recurrent polyarthralgia after a course of 15 months [7]. Consistently, the figure rose to 64% in hospitalized CHIK+ patients at 18 months post infection [6]. However, in all these studies, the causality between subjective rheumatic symptoms and CHIKV infection was questionable, either due to participation and selection biases, to absence of standardization in symptom elicitation, to lack of clinical review, and to the absence of an uninfected control group to assess symptom rates at baseline.

Infections with other 'arthritogenic' alphaviruses such as RRV or Sindbis-related (Pogosta) virus were associated with same magnitude of incidence (50% to 60%) of persisting arthralgia 6 months to 3 years after acute infection [40-43]. Interestingly, half of the cohort of RRV patients exhibited signs over time of chronic rheumatic disease [44]. Similarly, Borgherini *et al. *reported that 44% of Réunionese patients declaring prolonged Chikungunya arthritis had a previous history of joint pain [6]. Accordingly, Sissoko *et al. *identified the presence of underlying osteoarthritis (OA) comorbidity as a significant risk factor for non-recovery [7]. This is why the possibility of overlap between a rheumatic pre-existing underlying condition and an authentic Chikungunya-specific arthralgia led the more skeptical contributors to think that prolonged signs of 'arthritogenic' alphaviruses could represent the mode of revelation of chronic rheumatic diseases, such as RA or psoriatic rheumatism [6,23,24,42,43].

Our study has shown that among CHIKV infected individuals, 60% of polyarthralgia could be attributed to CHIKV. The understanding of the musculoskeletal symptoms of Chikungunya has taken a significant step forward with the recent development of a non-human primate model [10]. These data confirm the central place of the macrophage as the main target cell for CHIKV dissemination and persistence within the organism, as well as being the cornerstone for CHIKV rheumatic pathophysiology [8,11,12]. Thus, similarly to RRV or RA [9], an inadequate host innate immune (Th1) response to CHIKV could damage cartilage and be the source of OA degenerative lesions [45]. With regard to the potential community burden of Chikungunya-specific rheumatism, efforts should focus on the control of Chikungunya joint damage with pre-existing or newly developed anti-inflammatory drugs [9,24,46]. For these reasons, further research including randomized clinical trials are required to better understand the mechanisms of CHIKV pathophysiology and promote the prevention of the progression of OA in areas affected by this 'arthritogenic' virus.

The fact Chikungunya is responsible for so many issues, involved in 13.5% of fatigue states and in 24.2% of light cerebral disorders, is not surprising. On the one hand, CHIKV neurotropism is now well documented through various observations at all ages of life [15-19]. Moreover, there is a growing body of evidence for the presence of neuropathic pain in non-responsive CHIKV patients associated with a more aggressive clinical picture and more challenging treatment, which can impair quality of life and even lead to depression [47]. On the other hand, some evidence supports the putative role of the interferon family, and more widely of Th1 cytokines in primary and drug-induced sickness behavior syndromes [48]. Interestingly, the two conditions include tiredness and mood disturbance, both previously related to RRV through the postinfective fatigue syndrome [49,50]. Indeed, Th1 cytokines are known to preferentially target neurocircuits relevant to psychomotor activity (for example, basal ganglia) [51]. Whether fatigue and light cerebral disorders in the context of CHIKV disease could share a common neuropathogenesis involving the innate immune system with RRV and other alphaviruses [48], or represent the harmful consequences on psychomotor activity of a long-lasting disease, deserves further research. Nonetheless, there is undoubtedly potential for a significant community burden of CHIKV neurotropism that can no longer be ignored.

Little attention was paid to sensorineural impairment before the recent Chikungunya epidemic. Thus, no observational study has yet focused on ophthalmic and hearing involvements beyond the few case series reported from the Indian subcontinent [20,21]. Our data demonstrate clearly that sensorineural health can be affected during large-scale CHIKV outbreaks, through various complaints such as hearing difficulties and blurred vision, independently of other conditions such as senescence and diabetes mellitus. The basis of these sensorial symptoms is still poorly understood and might depict another facet of CHIKV neurotropism rather than a direct targeting of sensory cells *per se*.

Taken together, with regard to the other aspects of severe Chikungunya disease over the epidemic, such as maternal-fetal transmission [16], myocarditis, Guillain-Barré syndrome [18,19] or other atypical forms with increased background mortality [13,14], the toll of long-term disabilities reported herein supports enhanced virulence of the E1-A226V strain compared to other CHIKV strains involved in previous Asian outbreaks [52], which argues definitively for revisiting Chikungunya as a benign non-fatal illness.

Our study has some strengths and limitations. The investigator was blinded to the serological status of the participants to limit the propensity of classification bias (overestimation of symptoms among CHIK+ subjects). Though participants were interviewed long after the onset of the disease, only ongoing symptoms were considered to minimize recall bias. In contrast with previous studies, a control group was used for comparison and subjects were randomly selected through a population-based representative cohort [27]. Thus, there was no substantial participation bias favoring patients with severe illness unlike previous hospital-based studies (non-representative of the baseline of CHIK+ patients) [26]. The diagnostic accuracy was definitely efficient, as shown by low proportions of asymptomatic cases and false positives [26,27]. Finally, we applied population weights to account for the case-mix representativeness and minimize the imbalance produced by selecting more women and older participants. However, patients were not examined physically by clinicians either to rule out differential diagnoses or to assess and confirm the complaints. Moreover, health perceptions may have been disturbed after such a public health catastrophe [53]. These reasons may have skewed the estimation of some of the subjective symptoms assessed in this survey of perceived morbidity. This said, the role of subjectivity could be considered as negligible in this study except for fatigue, as shown by the sensitivity analysis restricted to true positives and true negatives. It is also unlikely that subjectivity or the knowledge of exposure changed the overall meaning of our findings owing to the small proportions of false positives and false negatives in the community [27], and of participants not knowing their serostatus enrolled in the TELECHIK survey. Nevertheless, it is important to emphasize that the disease burden in terms of common manifestations may be overstated by subjectivity and previous knowledge of exposure, especially for an emerging arboviral infection when it is associated with illness-specific catastrophic thinking [54]. Although this phenomenon seems to us to be relatively marginal, it cannot be ruled out because Chikungunya was ranked third (after AIDS and cancer) on the scale of health risk perceptions shortly after the outbreak (Perrau J et al., personal communication).

## Conclusions

On average, 2 years after onset of infection 43% to 75% of CHIK+ patients report prolonged or late-onset symptoms highly attributable to CHIKV. These manifestations carry a significant burden in the community in the fields of rheumatology, neurology and sensorineural health. Our findings lend support to clinical descriptions, pathophysiology, and perception of Chikungunya disease and give a valuable insight towards the understanding of the economic impact of this disabling arboviral disease.

## Competing interests

The authors declare that they have no competing interests.

## Authors' contributions

PG analyzed the data, drafted and reviewed the manuscript; AF helped to design the study, analyzed the data, reviewed the data for consistency and errors and also checked the manuscript; DM, AM, BAG, GB and FF reviewed the manuscript, each in his field of expertise, for consistency and perspective; CM performed telephone interviews, and entered the data; KB realized the data management; OR helped to analyze the data; AM performed the serology assays during the SEROCHIK survey; FF was the principal investigator of the SEROCHIK and TELECHIK surveys. All authors read and approved the final manuscript.

## Pre-publication history

The pre-publication history for this paper can be accessed here:

http://www.biomedcentral.com/1741-7015/9/5/prepub

## Supplementary Material

Additional file 1 **Distribution of the time elapsed between the onset of infection and the TELECHIK survey in seropositive (CHIK+) subjects. **The histogram displays the range of the time elapsed between the onset of infection and telephonic interviews in Chikungunya virus (CHIKV) infected subjects. Click here for file

Additional file 2 **Table S1. **Crude weighted rates and prevalence ratios of self-reported symptoms/Chikungunya virus (CHIKV) status from the TELECHIK survey, La Réunion Island population, November 2007 to May 2008. The weighted rates of the symptoms are expressed as percentages in parentheses; Chikungunya status is defined by specific anti-Chikungunya virus (CHIKV) IgG antibodies. TN = true negative (no self-reported Chikungunya disease with no infection confirmed by CHIKV-specific IgG antibodies); TP = true positive (self-reported Chikungunya disease with infection confirmed by CHIKV-specific IgG antibodies). Click here for file

Additional file 3 **Table S2. **Adjusted prevalence ratios for the determinants of self-reported rheumatic symptoms, TELECHIK survey, La Réunion Island population, November 2007 to May 2008. *Diabetes mellitus, hypertension, ischemic heart disease, asthma, chronic obstructive pulmonary disease, renal failure, cancer. TN = true negative (no self-reported Chikungunya disease with no infection confirmed by Chikungunya virus (CHIKV)-specific IgG antibodies); TP = true positive (self-reported Chikungunya disease with infection confirmed by CHIKV-specific IgG antibodies). Click here for file

Additional file 4 **Table S3. **Adjusted prevalence ratios for the determinants of light cerebral disorders, TELECHIK survey, La Réunion Island population, November 2007 to May 2008. *Osteoarthritis, diabetes mellitus, hypertension, ischemic heart disease, asthma, chronic obstructive pulmonary disease, renal failure, cancer. TN = true negative (no self-reported Chikungunya disease with no infection confirmed by Chikungunya virus (CHIKV)-specific IgG antibodies); TP = true positive (self-reported Chikungunya disease with infection confirmed by CHIKV-specific IgG antibodies). Click here for file

Additional file 5 **Table S4. **Adjusted prevalence ratios for the determinants of sensorineural impairment, TELECHIK survey, La Réunion Island population, November 2007 to May 2008. *Osteoarthritis, diabetes mellitus, hypertension, ischemic heart disease, asthma, chronic obstructive pulmonary disease, renal failure, cancer. TN = true negative (no self-reported Chikungunya disease with no infection confirmed by Chikungunya virus (CHIKV)-specific IgG antibodies); TP = true positive (self-reported Chikungunya disease with infection confirmed by CHIKV-specific IgG antibodies). Click here for file

Additional file 6 **Table S5. **Adjusted prevalence ratios for the determinants of fatigue, TELECHIK survey, La Réunion Island population, November 2007 to May 2008. *Osteoarthritis, diabetes mellitus, hypertension, ischemic heart disease, asthma, chronic obstructive pulmonary disease, renal failure, cancer. TN = true negative (no self-reported Chikungunya disease with no infection confirmed by Chikungunya virus (CHIKV)-specific IgG antibodies); TP = true positive (self-reported Chikungunya disease with infection confirmed by CHIKV-specific IgG antibodies). Click here for file
