# Supplementary figures and images for: Perceived morbidity and community burden after a Chikungunya outbreak: the TELECHIK survey, a population-based cohort study
Source: BMC Med. 2011 Jan 14;9:5. doi: 10.1186/1741-7015-9-5 (PMC3029216; doi:10.1186/1741-7015-9-5)

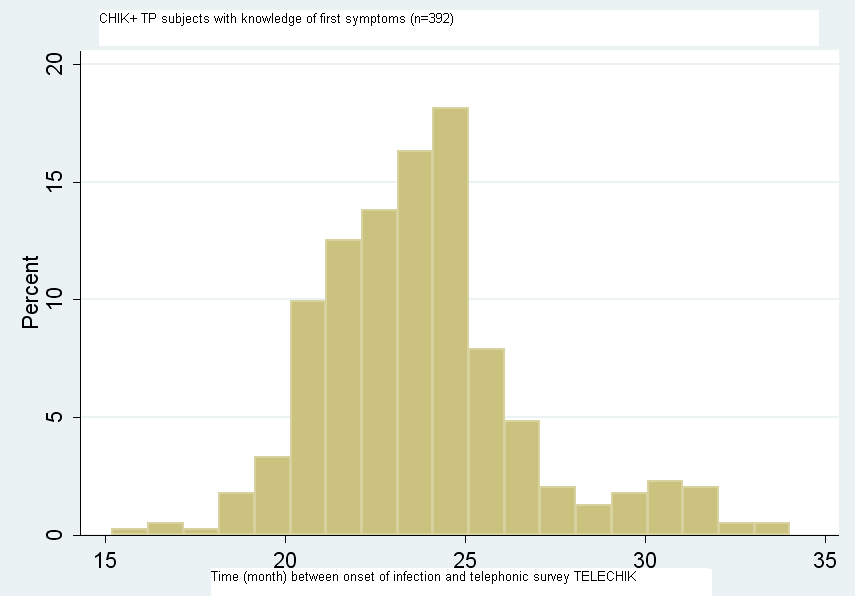

Supplement: Additional file 1 — Distribution of the time elapsed between the onset of infection and the TELECHIK survey in seropositive (CHIK+) subjects. The histogram displays the range of the time elapsed between the onset of infection and telephonic interviews in Chikungunya virus (CHIKV) infected subjects. [file 1741-7015-9-5-S1.TIFF]
