# Supplementary material for: Perceived morbidity and community burden after a Chikungunya outbreak: the TELECHIK survey, a population-based cohort study
Source: BMC Med. 2011 Jan 14;9:5. doi: 10.1186/1741-7015-9-5 (PMC3029216; doi:10.1186/1741-7015-9-5)
Supplement: Additional file 2 — Table S1. Crude weighted rates and prevalence ratios of self-reported symptoms/Chikungunya virus (CHIKV) status from the TELECHIK survey, La Réunion Island population, November 2007 to May 2008. The weighted rates of the symptoms are expressed as percentages in parentheses; Chikungunya status is defined by specific anti-Chikungunya virus (CHIKV) IgG antibodies. TN = true negative (no self-reported Chikungunya disease with no infection confirmed by CHIKV-specific IgG antibodies); TP = true positive (self-reported Chikungunya disease with infection confirmed by CHIKV-specific IgG antibodies). [file 1741-7015-9-5-S2.DOC]

| **Table S1. Crude weighted rates and prevalence ratios of self-reported symptoms×CHIKV status*** | | | | | | | | | | | |
| --- | --- | --- | --- | --- | --- | --- | --- | --- | --- | --- | --- |
| **TELECHIK survey, La Réunion Island population, November 2007 to May 2008** | | | | | | | | | | | |
| **Symptoms (%)** | CHIK - TN | **CHIK+ TP** | | **Crude PR** | | | **(95% CI)** | | | **P value** | |
| **Musculoskeletal pain** | (14.1) | (45.2) | | | 3.2 | | | (2.4 – 4.3) | | | < 0.001 |
| **Fatigue** | (44.7) | (56.7) | | | 1.3 | | | (1.1 – 1.5) | | | 0.003 |
| **Light cerebral disorders** | (53.6) | (77.6) | | | 1.4 | | | (1.2 – 1.6 ) | | | < 0.001 |
| Headache | (16.2) | (27.1) | | | 1.7 | | | (1.2 – 2.3) | | | 0.001 |
| Sleep disorders | (22.5) | (33.4) | | | 1.5 | | | (1.1 – 1.9) | | | 0.003 |
| Memory troubles | (22.8) | (44.3) | | | 1.9 | | | (1.5 – 2.4) | | | < 0.001 |
| Attention difficulties | (17.6) | (39.7) | | | 2.3 | | | (1.7 – 3.0) | | | < 0.001 |
| Mood disturbance | (22.6) | (38.6) | | | 1.7 | | | (1.3 – 2.2) | | | < 0.001 |
| Depression | (6.7) | (14.9) | | | 2.2 | | | (1.3 – 3.6) | | | 0.001 |
| **Sensorineural** | (34.6) | (51.8) | | | 1.5 | | | (1.2 – 1.8) | | | < 0.001 |
| Blurred vision | (27.5) | (44.6) | 1.6 | | | (1.3 – 2.0) | | | < 0.001 | | |
| Hearing difficulties | (12.0) | (18.9) | 1.6 | | | (1.1 – 2.3) | | | 0.016 | | |
| **Digestive** | (13.0) | (18.6) | 1.4 | | | (0.9 – 2.1) | | | 0.06 | | |
| **Dermatologic** | (32.2) | (38.0) | 1.2 | | | (0.9 – 1.5) | | | 0.13 | | |
| Skin lesion | (16.7) | (20.4) | 1.2 | | | (0.8 – 1.7) | | | 0.23 | | |
| Alopecia | (19.0) | (24.1) | 1.3 | | | (0.9 – 1.7) | | | 0.11 | | |
| The weighted rates of the symptoms are expressed as percentages in parentheses; *Chikungunya status is defined by specific anti-CHIKV IgG antibodies;  TP: true positive (self-reported Chikungunya disease with infection confirmed by CHIKV-specific IgG antibodies); TN: true negative (no self-reported Chikungunya disease with no infection confirmed by CHIKV-specific IgG antibodies) | | | | | | | | | | | |
