# Supplementary material for: Perceived morbidity and community burden after a Chikungunya outbreak: the TELECHIK survey, a population-based cohort study
Source: BMC Med. 2011 Jan 14;9:5. doi: 10.1186/1741-7015-9-5 (PMC3029216; doi:10.1186/1741-7015-9-5)
Supplement: Additional file 3 — Table S2. Adjusted prevalence ratios for the determinants of self-reported rheumatic symptoms, TELECHIK survey, La Réunion Island population, November 2007 to May 2008. *Diabetes mellitus, hypertension, ischemic heart disease, asthma, chronic obstructive pulmonary disease, renal failure, cancer. TN = true negative (no self-reported Chikungunya disease with no infection confirmed by Chikungunya virus (CHIKV)-specific IgG antibodies); TP = true positive (self-reported Chikungunya disease with infection confirmed by CHIKV-specific IgG antibodies). [file 1741-7015-9-5-S3.DOC]

| **Table S2. Adjusted prevalence ratios for the determinants of self-reported rheumatic symptoms,** | | | |
| --- | --- | --- | --- |
| **TELECHIK survey, La Réunion Island population, November 2007 to May 2008** | | | |
| **Determinants** | Adjusted PR | **(95% CI)** | **P value** |
| **Chikungunya** |  |  | < 0.001 |
| TN | 1 |  |  |
| TP | 2.6 | (1.9 – 3.4) |  |
| **Gender** |  |  | 0.195 |
| Male | 1 |  |  |
| Female | 1.2 | (0.9 - 1.5) |  |
| **Age (years)** |  |  | < 0.001 |
| < 20 | 1 |  |  |
| 20 to 29 | 1.6 | (0.8 - 3.1) |  |
| 30 to 39 | 2.0 | (1.1 - 3.7) |  |
| 40 to 49 | 3.0 | (1.7 - 5.2) |  |
| 50 to 59 | 3.5 | (2.0 - 6.0) |  |
| 60 to 69 | 4.3 | (2.5 - 7.3) |  |
| ≥ 70 | 3.7 | (2.1 - 6.5) |  |
| **Body mass Index (kg/m^2^)** | |  | 0.028 |
| < 25 | 1 |  |  |
| 25 – 29.9 | 1.4 | (1.1 - 1.8) |  |
| ≥ 30 | 1.2 | (0.8 - 1.7) |  |
| **Comorbidity** |  |  | 0.252 |
| None | 1 |  |  |
| Osteoarthritis | 1.4 | (0.9 - 2.2) |  |
| Other* | 1.2 | (0.9 - 1.5) |  |
| *Diabetes mellitus, hypertension, ischemic heart disease, asthma, chronic obstructive pulmonary  disease, renal failure, cancer; TP: true positive (self-reported Chikungunya disease with infection confirmed by CHIKV-specific IgG antibodies); TN: true negative (no self-reported Chikungunya disease with no infection confirmed by CHIKV-specific IgG antibodies) | | | |
