# Supplementary material for: Perceived morbidity and community burden after a Chikungunya outbreak: the TELECHIK survey, a population-based cohort study
Source: BMC Med. 2011 Jan 14;9:5. doi: 10.1186/1741-7015-9-5 (PMC3029216; doi:10.1186/1741-7015-9-5)
Supplement: Additional file 4 — Table S3. Adjusted prevalence ratios for the determinants of light cerebral disorders, TELECHIK survey, La Réunion Island population, November 2007 to May 2008. *Osteoarthritis, diabetes mellitus, hypertension, ischemic heart disease, asthma, chronic obstructive pulmonary disease, renal failure, cancer. TN = true negative (no self-reported Chikungunya disease with no infection confirmed by Chikungunya virus (CHIKV)-specific IgG antibodies); TP = true positive (self-reported Chikungunya disease with infection confirmed by CHIKV-specific IgG antibodies). [file 1741-7015-9-5-S4.DOC]

| **Table S4. Adjusted prevalence ratios for the determinants of light cerebral disorders,** | | | |
| --- | --- | --- | --- |
| **TELECHIK survey, La Réunion Island population, November 2007 to May 2008** | | | |
| **Determinants** | Adjusted PR | **(95% CI)** | **P value** |
| **Chikungunya** |  |  | < 0.001 |
| TN | 1 |  |  |
| TP | 1.4 | (1.2 – 1.6) |  |
| **Gender** |  |  | 0.019 |
| Male | 1 |  |  |
| Female | 1.2 | (1.0 - 1.3) |  |
| **Age (years)** |  |  | 0.76 |
| < 20 | 1 |  |  |
| 20 to 29 | 1.1 | (0.8 – 1.3) |  |
| 30 to 39 | 0.9 | (0.7 – 1.2) |  |
| 40 to 49 | 1.0 | (0.8 – 1.2) |  |
| 50 to 59 | 1.1 | (0.9 – 1.3) |  |
| 60 to 69 | 1.1 | (0.8 - 1.3) |  |
| ≥ 70 | 0.9 | (0.7 – 1.2) |  |
| **Comorbidity*** |  |  | 0.317 |
| None | 1 |  |  |
| One | 1.1 | (0.9 - 1.3) |  |
| Two | 1.2 | (0.9 - 1.4) |  |
| Three or more | 1.1 | (0.8 – 1.5) |  |
| *Osteoarthritis, diabetes mellitus, hypertension, ischemic heart disease, asthma, chronic obstructive pulmonary disease, renal failure, cancer;  TP: true positive (self-reported Chikungunya disease with infection confirmed by CHIKV-specific IgG antibodies); TN: true negative (no self-reported Chikungunya disease with no infection confirmed by CHIKV-specific IgG antibodies) | | | |
